# Effect of novel carboxymethyl cellulose-based dressings on acute wound healing dynamics

**DOI:** 10.17221/89/2023-VETMED

**Published:** 2023-10-30

**Authors:** Alzbeta Kruzicova, Marta Chalupova, Gabriela Kuzminova, Tomas Parak, Jarmila Klusakova, Tomas Sopuch, Pavel Suchy

**Affiliations:** ^1^Department of Human Pharmacology and Toxicology, Faculty of Pharmacy, University of Veterinary and Pharmaceutical Sciences in Brno, Brno, Czech Republic; ^2^Department of Pharmacology and Toxicology, Faculty of Pharmacy, Masaryk University, Brno, Czech Republic; ^3^Histos, Ltd., Brno, Czech Republic; ^4^Holzbecher, Ltd. – Bleaching & Dyeing Plant, Zlic, Czech Republic

**Keywords:** cytokines, growth factors, wound dressing, Wistar rat

## Abstract

The clinical implications and efficacy of newly developed modified cellulose materials were evaluated in an acute wound animal model. In the current study, sixty male rats were divided into four groups. A full-thickness circular excision wound was created in the suprascapular area. Newly developed matrices (acidic partially carboxymethylated cellulose; acidic partially carboxymethylated cellulose impregnated with a povidone-iodine solution) were applied in two test groups, while fifteen animals were used as a control group without any primary dressing. Aquacel Ag, a clinically used dressing, was selected as the reference material. To compare the efficacy *in vivo*, the wound size and production of selected cytokines and growth factors (TNF-α, TGF-β1, and VEGF), which play a key role in the healing process, were measured at two, seven, and fourteen days after surgery. The activity of matrix metalloproteinases 2 and 9, which actively participate in cell signalling and are essential for tissue remodelling, was determined in wound tissue by gelatin zymography. A positive effect of the newly developed dressing materials on the healing process, tissue granulation, and wound re-epithelialisation was demonstrated.

The primary goal of wound healing is to achieve rapid wound closure and the formation of a functional scar. Carboxymethyl cellulose, an anionic cellulose derivative containing the carboxymethyl functional group, has gained increasing popularity in wound treatment and surgery. This chemical modification provides a material with favourable properties (e.g., formation of colloidal systems in water-hydrocolloid fibres) that allows moist wound healing, facilitates a faster granulation and epithelisation process, and generally shortens the healing period.

Effective and uncomplicated wound healing requires the synergy of a number of cytokines and growth factors, which may have specific or pleiotropic functions, and their production is often associated with a particular wound-healing phase. The inflammatory phase begins as soon as the wound is formed and is considered the preparatory process that facilitates the formation of new tissue. During platelet degranulation, the platelet-derived growth factor (PDGF) and transforming growth factor-beta (TGF-β) are released (Kim et al. 1998). Wounds treated with neutralising antibodies to TGF-β1 have a reduced inflammatory response, reduced early extracellular matrix deposition, and reduced later cutaneous scarring, indicating the importance of local tissue TGF-β1 (Shah et al. 1995).

Tumour necrosis factor alpha (TNF-α) is an important mediator in the pathogenesis of trauma, sepsis, and inflammation. The intravascular application of TNF-α leads to capillary leakage and shock syndrome, whereas extravascular the application induces angiogenesis. The angiogenic effect of macrophages is mainly mediated by TNF-α (Leibovich et al. 1987; Sunderkotter et al. 1990; Sunderkotter et al. 1991; Rappolee and Werb 1992).

Local changes in the wound microenvironment, such as low pH, reduced oxygen tension, and elevated lactate levels, actually induce the release of factors necessary for the new blood supply (Knighton et al. 1983; LaVan and Hunt 1990). This process, called angiogenesis or neovascularisation, is stimulated by the vascular endothelial growth factor (VEGF). Other angiogenic growth factors, such as the basic fibroblast growth factor (bFGF) and transforming growth factor β (TGF-β) have been described (Battegay 1995; Tonnesen et al. 2000). However, VEGF is unique in its effects on multiple components of the wound healing cascade, including angiogenesis and, the more recently described, epithelisation and collagen deposition (Stojadinovic et al. 2007).

Cell migration, angiogenesis, degradation of the provisional matrix, and remodelling of the newly formed granulation tissue require controlled degradation of the extracellular matrix (ECM). Disruption of the balance between ECM production and degradation leads to the formation of chronic ulcers with excessive ECM degradation or fibrosis (Ravanti and Kahari 2000). Matrix metalloproteinases (MMPs) are a family of zinc-dependent endopeptidases that can degrade ECM components. The expression of MMPs by immune cells is strongly modulated by inflammatory mediators, such as TNF-α, IL-1β, and IL-4 (Mauviel 1993; Birkedal-Hansen 1995). Although MMPs play an important role, their presence in the wound bed for too long and/or in an excessive amount leads to the degradation of non-natural substrates, such as growth factors, receptors, and ECM proteins, that are necessary for physiological healing and their insufficiency can lead to the development of chronic and non-healing wounds. Therefore, neutralisation of MMPs may be beneficial for the treatment efficacy (Krejner and Grzela 2015). Several attempts have been made to reduce the protease activity by interfering with MMP expression or by designing smart wound dressings (Eming et al. 2008). To this end, materials with the addition of tissue inhibitors of metalloproteinases (TIMPs) and materials that lower the pH in the wound (e.g., acidic partially carboxymethylated cellulose) can be used. Nano or biogenic silver-containing materials also have the ability to reduce the relative mRNA and protein expression of MMP-2 and MMP-9 (Krishnan et al. 2018).

## MATERIAL AND METHODS

### Material

The tested matrices were acid partially carboxymethylated cotton cellulose (dressing name Hcel HT) in the form of spunlace non-woven fabric (HHT) and the same material impregnated with a povidone-iodine solution (PVP-I) at a ratio of 70.9 mg PVP per 100 cm^2^ (HTB), purchased from Holzbecher, Ltd. – Bleaching and Dyeing Plant, Zlic, Czech Republic. The sodium salt of the partially carboxymethylated regenerated cellulose containing 1.2% Ag in ionic form (wound dressing Aquacel Ag) in the form of needle-punched non-woven fabric (AAG) was used as the reference material. In the control group, the wound was covered only with a secondary dressing (sterile gauze).

### Animal model

Sixty male Wistar rats (180–220 g) were purchased from AnLab, Ltd. (Prague, Czech Republic) and kept under standard conditions (temperature 22 ± 2 °C, relative humidity 50 ± 10%, 12-hour alternating light/dark cycle). The rats were given a standard diet and water *ad libitum.* The experimental protocol was approved by the Expert Committee for the Welfare of Experimental Animals of the University of Veterinary and Pharmaceutical Sciences Brno, Czech Republic. General anaesthesia was induced in the animal by intramuscular injection of Zoletil at a dose of 65 mg/kg. A full-thickness circular skin wound, 15 mm in diameter, was created under general anaesthesia. The tested materials, reference dressing, and secondary dressing (sterile gauze) were applied to the wound; in the control group, only the secondary dressing was applied. A total of 60 animals were divided into four groups of 15 animals each. One-third of the animals in each group were euthanised by the intracardiac injection of the T61 agent at two, seven, and fourteen days after surgery. The wound size (diameter) was measured and a tissue sample was collected for the western blot, zymography, and histologic examinations.

### Histology

Tissue samples were fixed in 10% neutral buffered formalin at room temperature, dehydrated through a graded alcohol bath (30–100%) and embedded in paraffin. Tissue sections (6 μm) were stained with haematoxylin-eosin. The tissue samples for histologic examination were collected at two-, seven-, and fourteen-day intervals. A total of five samples from each group were differentially diagnosed fourteen days after surgery: the wound epithelised surface (in percent) and the presence of a granulomatous inflammatory reaction (scale 0 – no incidence, 1 – mild incidence, 2 – medium incidence to 3 – massive presence). The mean value was calculated for each group.

### Western blot

Using a bench blender, approximately 0.1 g of wound tissue was homogenised in a lysis buffer [50 mM Tris-HCl (pH 7.5), 1 mM ethylene glycol-bis(β-aminoethyl ether)-N,N,N',N'-tetraacetic acid (EGTA), 1 mM ethylenediaminetetraacetic acid (EDTA), 1 mM sodium orthovanadate, 50 mM sodium fluoride, 5 mM sodium pyrophosphate, 0.27 M saccharose] at a ratio of 3 ml of buffer per gram tissue. After centrifugation at 13 444 ×* g* for 15 min at 4 °C, the supernatant was stored. The protein concentration was measured using a Bradford method protein assay kit (Amresco, Solon, USA) according to the manufacturer’s instructions. The protein samples were denatured in the presence of β-mercaptoethanol and sodium dodecyl sulfate (SDS) at 90 °C for 5 minutes. Fifty micrograms (50 μg) of the total protein were loaded on a 12% SDS-polyacrylamide gel. After electrophoresis (140 V, 400 mA, 90 min), the proteins were transferred to a nitrocellulose membrane (0.2 μm; Bio-Rad, Hercules, USA), which was then blocked for one hour with 5% Bovine Serum Albumin (BSA) (Sigma-Aldrich, St. Louis, MO, USA) in a Tris Buffered Saline with Tween 20 (TBST) buffer [10 mM Tris-HCl (pH 7.5), 150 mM sodium chloride, 0.1% (v/v) Tween-20]. The membrane was then incubated with a primary rabbit anti-TNF-α antibody at a 1 : 2 000 dilution (Abcam, Cambridge, UK), a mouse anti-TGF-β1 antibody at a 1 : 2 000 dilution (Abcam, Cambridge, UK), a rabbit anti-VEGF antibody at a 1 : 7 500 dilution (Abcam, Cambridge, UK) overnight at 4 °C. After washing with distilled water, the membrane was incubated with a secondary antibody (anti-mouse IgG or anti-rabbit IgG; Sigma-Aldrich, St. Louis, MO, USA) at a dilution of 1 : 2 000 for one hour at room temperature. The secondary antibody was detected by colorimetric analysis using an Opti-CNTM Substrate Kit (Bio-Rad, Hercules, USA). The intensity of the protein bands was evaluated by densitometric analysis using Image Studio^TM^ Lite (LI-COR Biotechnology, Lincoln, USA).

### Gelatin zymography

The MMP activity (MMP-2, MMP-9) was evaluated using gelatin zymography. Five micrograms of native proteins (without denaturation in the presence of β-mercaptoethanol) were loaded onto a 10% SDS-polyacrylamide gel impregnated with 0.1% gelatin. After electrophoresis (140 V, 400 mA, 90 min), the gel was washed twice for 15 min in 2.5% (v/v) Triton X-100 to remove the SDS. The gel was then incubated for 15 min at room temperature and subsequently overnight (about 1 h) at 37 °C in the development buffer [50 mM Tris-HCl (pH 8.8), 5 mM calcium chloride, 3 mM sodium azide, 0.5% (v/v) Triton X-100]. After that, the gel was stained with Coomassie blue for 2 h and destained until the bands were clearly visible. The intensity of the digested bands was determined by densitometric analysis using Image Studio^TM^ Lite (LI-COR Biotechnology, Lincoln, USA).

### Statistical analysis

The results are expressed as mean values, with error bars representing the standard error (SE) of the mean. A one-way analysis of variance (ANOVA) was used for the statistical evaluation (Microsoft Excel; Microsoft Corporation, USA). Values of *P* < 0.05 were considered to be statistically significant, and values of *P* < 0.01 were considered to be highly statistically significant compared to the control group.

## RESULTS

### Wound size and histology

Two days after the circular full-thickness skin excision, no significant differences in the wound size were observed with the tested substances compared to the control group (Table 1).

**Table 1:** Wound size two, seven, and fourteen days after the circular full-thickness skin excision in the suprascapular area

Dressing	Wound size (mm)		
	2 days	7 days	14 days
CON	13.0 ± 1.22	9.60 ± 2.70	4.60 ± 2.50
HHT	14.20 ± 0.84	10.40 ± 2.19	4.80 ± 2.28
HTB	13.80 ± 0.45	11.40 ± 0.89	4.80 ± 3.11
AAG	12.0 ± 1.0	10.40 ± 1.34	5.80 ± 2.28

AAG = Aquacel Ag; CON = control group; HHT = Hcel HT; HTB = Hcel HT impregnated with the povidone-iodine solution

After seven days, the smallest wound size was observed in the control group (9.6 ± 2.70 mm). The tested material Hcel HT and the reference dressing Aquacel Ag achieved similar results (10.40 ± 2.19 mm and 10.40 ± 1.34 mm, respectively), but there are no statistically significant differences compared to the control group. After, the minor wound diameter was measured in the control group (4.60 ± 2.50 mm), and the matrices Hcel HT (4.80 ± 2.28 mm) and Hcel HT with the povidone-iodine solution (4.80 ± 3.11 mm) were comparable with this result.

The percentage of the newly epithelised wound surface and the presence of a granulomatous inflammatory reaction were evaluated fourteen days after surgery, and the results are summarised in Table 2.

**Table 2:** Acute skin wound re-epithelisation (percentage of epithelised surface) and the presence of a granulomatous reaction fourteen days after surgery

Dressing	Epithelised surface (%)	Granulomatous reaction
CON	64 ± 26.1	1.6 ± 0.89
HHT	100 ± 0*	0.4 ± 0.55*
HTB	51 ± 43.1	1.6 ± 1.52
AAG	89.2 ± 24.2	0.17 ± 0.41**

AAG = Aquacel Ag; CON = control group; HHT = Hcel HT; HTB = Hcel HT impregnated with the povidone-iodine solution

*Values of *P* < 0.05 were considered to be statistically significant; **Values of *P* < 0.01 were considered to be highly statistically significant compared to the control group

Scale 0 – no incidence, 1– mild incidence, 2 – medium incidence, 3 – massive presence

**Figure 1:**
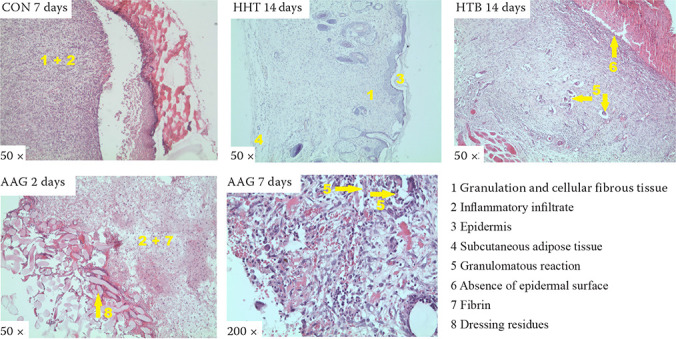
Wound tissue sections from the control group, tested materials and the reference dressing, haematoxylin-eosin stained, magnification fifty times to two hundred times AAG = Aquacel Ag; CON = control group; HHT = Hcel HT; HTB = Hcel HT impregnated with the povidone-iodine solution

Although the control group achieved the best results in the wound size after both seven and fourteen days, the histological examination shows that the wound surface was incompletely epithelised (only 64%). There was still a very pronounced acute component of fibrin, inflammatory exudate, and granulation tissue with newly formed tissue at the base of the wound (Figure 1). In addition, there was, together with the HTB group, the highest incidence of a granulomatous reaction (score 1.6). In contrast, the HHT group achieved complete wound re-epithelisation (100%, *P* = 0.015) with a low presence of chronic inflammatory infiltrate cells and no significant granulomatous reaction (score 0.4, *P* = 0.034) compared to the control group. In the wound treated with the reference material AAG, two days after surgery, residue of the dressing is visible on the surface of the wound, the epidermis is missing and an oedema with fibrin exudation and cells of the inflammatory infiltrate are under the material (Figure 1).

At seven days, the presence of an inflammatory exudate and an incipient granulomatous reaction are evident.

Although the material remains in the tissue at fourteen days, the wound was 89.2% re-epithelised and the granulomatous reaction score (0.17) was highly statistically significant (*P* = 0.006) compared to the control group.

### Wound healing dynamics

**Figure 2:**
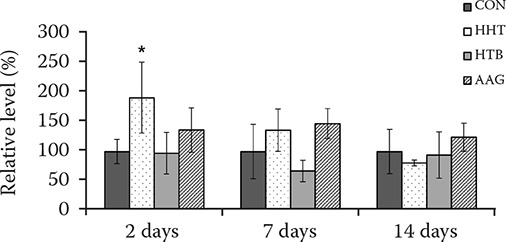
Relative levels of tumour necrosis factor alpha (TNF-α) *Values of *P* < 0.05 were considered to be statistically significant compared to the control group AAG = Aquacel Ag; CON = control group; HHT = Hcel HT; HTB = Hcel HT impregnated with the povidone-iodine solution

Changes in the levels of selected cytokines and growth factors accompanying the individual healing phases were monitored. Two days after surgery, the acidic carboxymethyl cellulose HHT statistically significantly (*P* = 0.012) elevated the level of tumour necrosis factor-alpha TNF-α to 192% compared to the control group, which was considered 100% (Figure 2).

The reference material AAG also had such an effect and increased the TNF-α level (137%), but the difference was not statistically significant compared to the control group. Seven days after surgery, this trend continued in the test HHT and reference AAG groups, in contrast to the HTB dressing, which caused a decrease in the relative amount of TNF-α (66.8%). After fourteen days, the acid carboxymethyl cellulose dressing HHT reduces the TNF-α levels (80.5%) because the wound is already completely re-epithelised (Table 2) and the signalling pathways associated with inflammation are suppressed. On the other hand, AAG maintains the trend of increasing the pro-inflammatory cytokine levels at all the time intervals.

**Figure 3:**
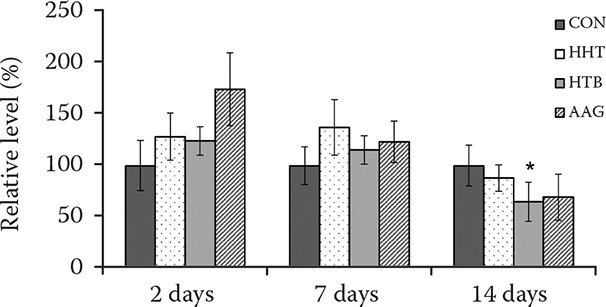
Relative levels of the transforming growth factor beta 1 (TGF-β1) *Values of *P* < 0.05 were considered to be statistically significant; **Values of *P* < 0.01 were considered to be highly significant compared to the control group AAG = Aquacel Ag; CON = control group; HHT = Hcel HT; HTB = Hcel HT impregnated with the povidone-iodine solution

The transforming growth factor-beta 1 (TGF-β1) plays a pivotal role in the granulation phase of wound healing and was chosen as a marker for the formation of granulation tissue that will gradually fill the wound. As shown in Figure 3, its relative levels were increased in all the test and reference groups two days after surgery, with the AAG dressing (175%) showing a highly significant difference (*P* = 0.004) compared to the control group.

This phenomenon is observed even on the seventh day and is related to the formation and deposition of collagen fibres. On the other hand, the TGF-β1 levels decreased in all the groups after fourteen days and the HTB group (64.5%) reached a significant difference (*P* = 0.02) compared to the control group.

**Figure 4:**
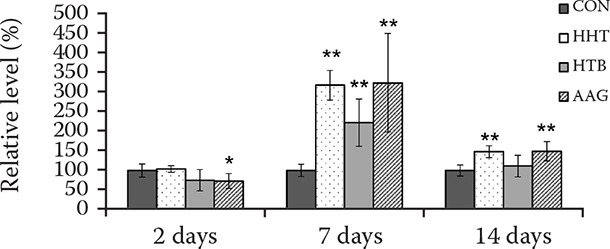
Relative levels of the vascular endothelial growth factor (VEGF) *Values of *P* < 0.05 were considered to be statistically significant; **Values of *P* < 0.01 were considered to be highly significant compared to the control group AAG = Aquacel Ag; CON = control group; HHT = Hcel HT; HTB = Hcel HT impregnated with the povidone-iodine solution

Angiogenesis is stimulated by the vascular endothelial cell growth factor (VEGF). The levels of this important growth factor were highly significantly affected by the tested matrices and also by the reference dressing, especially on day seven after skin excision (Figure 4).

At fourteen days, the levels were highly significantly increased in the HHT-tested group (149%) and also in the AAG group treated with the reference dressing (150%).

**Figure 5:**
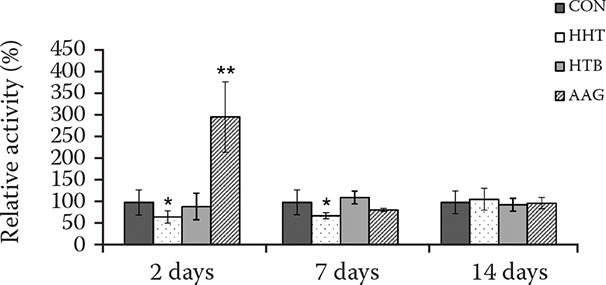
Relative activity of matrix metalloproteinase 2 (MMP-2) *Values of *P* < 0.05 were considered to be statistically significant; **Values of *P* < 0.01 were considered to be highly significant compared to the control group AAG = Aquacel Ag; CON = control group; HHT = Hcel HT; HTB = Hcel HT impregnated with the povidone-iodine solution

The effect of the novel dressings on the matrix metalloproteinase activity was investigated. The relative activity of MMP-2 was significantly reduced (Figure 5) by the HHT dressing at two days (65.6%, *P* = 0.047) and seven days (68.7%, *P* = 0.049) after surgery due to the acidic nature of the material.

**Figure 6:**
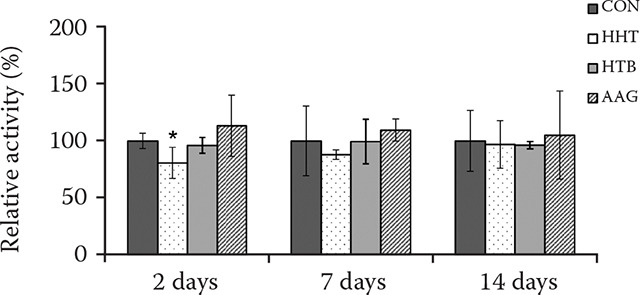
Relative activity of matrix metalloproteinase 9 (MMP-9) *Values of *P* < 0.05 were considered to be significant compared to the control group AAG = Aquacel Ag; CON = control group; HHT = Hcel HT; HTB = Hcel HT impregnated with the povidone-iodine solution

Surprisingly, the reference dressing AAG, increased the MMP-2 activity three times (*P* = 0.000 9) on day two compared to the control group (100%). The obtained data indicate that the activity of MMP-9 was less affected (Figure 6), but the same trend of a reduction in the activity due to the character of the dressing material is observed in the HHT group on the second (80.6%, *P* = 0.02) and seventh (87.9%) days after surgery.

## DISCUSSION

The best result in the wound size parameter was achieved by the control group (4.60 ± 2.50 mm), but the tested matrices Hcel HT (4.80 ± 2.28 mm) and Hcel HT with the povidone-iodine solution (4.80 ± 3.11 mm) were comparable with this result. Hcel HT is a modified carboxymethylated cellulose fibrous textile material characterised by improved properties, such as increased absorbency and sorption capacity, biocompatibility without skin irritation, and rapid haemostasis. Carboxymethyl cellulose-based materials have been shown to be effective against bleeding. In a recent study, a carboxymethyl cellulose textile achieved a significantly shorter time to haemostasis than the reference oxycellulose material (Suchy et al. 2020). The haemostatic effect of this material and the reduction of necrosis and interstitial haemorrhages with regenerative processes in the parenchyma were described by Chalupova et al. (2012). The positive effect of cellulose materials on acute skin wound healing in healthy rats and rats with induced diabetes mellitus was confirmed in a study by Basu et al. (2018). Wound contraction alone is not indicative of the quality of healing and tissue remodelling processes; therefore, the degree of wound surface epithelisation and the presence of a granulomatous reaction were also monitored. Complete re-epithelisation (100%) was achieved in the HHT group 14 days after surgery, with a low granulomatous reaction rate (0.4). In another study, results on the wound healing efficacy of carboxymethyl cellulose (CMC) scaffolds suggested that these scaffolds demonstrated a reduction in the partial-thickness wound size and achieved complete re-epithelisation in 22 days (Ramli and Wong 2011). The acid partially carboxymethylated cellulose Hcel HT statistically significantly increased the level of tumour necrosis factor-alpha TNF-α to 192% compared to the control group two days after surgery and similarly increased the relative level of the transforming growth factor-beta 1 (TGF-β1) over a seven day interval. This change in growth factor dynamics resulted in the promotion of the granulation phase of wound healing, as the production of most of these key extracellular matrix (ECM) components, such as collagens and fibronectin, is promoted in part by PDGF and TGF-β1 (Pakyari et al. 2013). TGF-β1 also inhibits the synthesis of MMPs and results in the greater accumulation of collagen fibres. All the tested materials and the reference dressing showed a highly significant increase in the relative amount of VEGF at 7 and 14 days (except for the HTB group at 14 days). This may be another mechanism by which carboxymethyl cellulose materials accelerate neovascularisation and healing, as VEGF is responsible for angiogenesis in wounds by triggering the angiogenic cascade (White et al. 2021) and promoting epithelialisation and collagen deposition (Stojadinovic et al. 2007). In a mouse model, Rossiter et al. (2004) confirmed that the absence of a keratinocyte-specific VEGF is responsible for the aberrant angiogenesis and delayed wound healing. Other studies have confirmed that reduced angiogenesis plays a prominent role in the non-healing nature of diabetic foot ulcers and other chronic wounds (Brem and Tomic-Canic 2007).

Matrix metalloproteinases (MMPs) are present in both acute and chronic wounds. They and their inhibitors play a critical role in regulating the degradation and deposition of extracellular matrix, which is essential for wound epithelisation. Excessive protease activity can lead to chronic non-healing wounds. The timed expression and activation of MMPs in response to wounding is critical for successful wound healing (Caley et al. 2015). The relative activities of MMP-2 and MMP-9 were measured by gelatin zymography two, seven, and fourteen days after circular skin excision. The Hcel HT dressing statistically significantly reduced the MMP-2 activities at two days (65.6%, *P* = 0.047) and seven days (68.7%, *P* =0.049) after surgery. This phenomenon can be explained by the acidic nature of the novel dressing material, as MMPs show maximum activity at an alkaline pH of ~ 8. The MMP-2 enzyme is dynamically influenced by the pH: at a low pH, the extracted enzyme activates a latent form, whereas collagen degradation by the matrix-bound enzyme is only observed at a pH close to neutral (Amaral et al. 2018). In a study by Khajah et al. (2013), the authors demonstrated an increased MMP-2/9 activity in response to the elevated extracellular pH. Surprisingly, the reference dressing AAG increased the MMP-2 activity three times (*P* = 0.000 9) on day 2 compared to the control group (100%). The data contrast with studies in which silver nanoparticles caused a reduction in the local matrix metalloproteinase (MMP) activity, thereby reducing inflammation (Kirsner et al. 2001). However, a histological examination of the wound tissue showed persistent inflammation and oedema with fibrin exudation under the dressing in the AAG group two days after surgery. In addition, material residues are present in the tissue; therefore, the MMP-2 activity may be increased to allow ECM remodelling (Kandhwal et al. 2022). As shown in Figure 6, the MMP-9 activity was not significantly affected, which correlates with the study that suggests that scaffold degradation is mainly caused by MMP-2, whereas inflammation increases the levels of this molecule and is not associated with an increase in MMP-9 (Nessler et al. 2014). Nanocrystalline silver dressings significantly reduced the MMP-9 levels and improved wound healing in a porcine model, although no mechanism was proposed (Wright et al. 2002).

The newly developed carboxymethyl cellulose-based dressing materials were shown to have a beneficial effect on the healing process, tissue granulation, and wound re-epithelialisation. The levels of the cytokine TNF-α and the growth factor TGF-β1 were affected, and the data showed a significant effect on the vascular endothelial growth factor. At the same time, the ability of the acidic partially carboxymethylated cellulose dressing Hcel HT to modulate the activity of the matrix metalloproteinases was observed. This demonstrated that dermal regeneration utilises molecular mechanisms and can be effectively promoted by the selection of an advanced wound dressing biomaterial.
